# The Overall Anxiety Severity and Impairment Scale as an Outcome Measure in Internet-Delivered Cognitive Behavioral Therapy for Anxiety Disorders: Observational Study

**DOI:** 10.2196/45362

**Published:** 2023-08-17

**Authors:** Boris Karpov, Jari Olavi Lipsanen, Ville Ritola, Tom Rosenström, Suoma Saarni, Satu Pihlaja, Jan-Henry Stenberg, Paula Laizane, Grigori Joffe

**Affiliations:** 1 Department of Psychiatry Helsinki University Hospital Helsinki Finland; 2 Finnish Psychological Association Helsinki Finland; 3 Department of Psychology and Logopedics Faculty of Medicine University of Helsinki Helsinki Finland; 4 Department of Psychiatry Helsinki University Hospital University of Helsinki Helsinki Finland; 5 Riga Stradins University Riga Latvia

**Keywords:** Overall Anxiety Severity and Impairment Scale, OASIS, internet-delivered cognitive behavioral therapy, iCBT, anxiety, social anxiety disorder, panic disorder, obsessive-compulsive disorder, OCD

## Abstract

**Background:**

Internet-delivered cognitive behavioral therapy (iCBT) is effective in the treatment of anxiety disorders. iCBT clinical trials use relatively long and time-consuming disorder-specific rather than transdiagnostic anxiety measurements. Overall Anxiety Severity and Impairment Scale (OASIS) is a brief self-report scale that could offer a universal, easy-to-use anxiety measurement option in disorder-specific and transdiagnostic iCBT programs.

**Objective:**

We aimed to investigate relationships between OASIS and disorder-specific instruments in iCBT. We expected these relationships to be positive.

**Methods:**

We investigated patients in original nationwide iCBT programs for generalized anxiety disorder (GAD), obsessive-compulsive disorder, panic disorder, and social anxiety disorder, which were administered by Helsinki University Hospital, Finland. In each program, anxiety symptoms were measured using both disorder-specific scales (the 7-item Generalized Anxiety Disorder scale, Penn State Worry Questionnaire, revised Obsessive-Compulsive Inventory, Panic Disorder Severity Scale, and Social Phobia Inventory) and by OASIS. A general linear model for repeated measures (mixed models) and interaction analysis were used for investigating the changes and relationships in the mean scores of OASIS and disorder-specific scales from the first session to the last one.

**Results:**

The main effect of linear mixed models indicated a distinct positive association between OASIS and disorder-specific scale scores. Interaction analysis demonstrated relatively stable associations between OASIS and the revised Obsessive-Compulsive Inventory (*F*_822.9_=0.09; 95% CI 0.090-0.277; *P*=.32), and OASIS and the Panic Disorder Severity Scale (*F*_596.6_=–0.02; 95% CI –0.108 to –0.065; *P*=.63) from first the session to the last one, while the 7-item Generalized Anxiety Disorder scale (*F*_4345.8_=–0.06; 95% CI –0.109 to –0.017; *P*=.007), Penn State Worry Questionnaire (*F*_4270.8_=–0.52; 95% CI –0.620 to –0.437; *P*<.001), and Social Phobia Inventory (*F*_862.1_=–0.39; 95% CI –0.596 to –0.187; *P*<.001) interrelated with OASIS more strongly at the last session than at the first one.

**Conclusions:**

OASIS demonstrates clear and relatively stable associations with disorder-specific symptom measures. Thus, OASIS might serve as an outcome measurement instrument for disorder-specific and plausibly transdiagnostic iCBT programs for anxiety disorders in regular clinical practice.

## Introduction

Anxiety disorders (ADs) are prevalent psychiatric conditions, with lifetime estimates of 16%-28% in the general population [1-3]. ADs have a substantial impact on health-related quality of life (HRQL), for example, affecting HRQL as much as those with heart failure [4]. Moreover, rates of health care use associated with medical conditions or psychiatric comorbidities are higher in patients with ADs than in those without anxiety. This also applies to reassurance-seeking behavior (ie, repeated demand of safety-related information with the purpose of reducing doubt or fear) in health-related anxiety [5,6]. Regardless of the high prevalence of ADs and notable unwanted impact on quality of life and health care service use, ADs often go unrecognized or are poorly treated [7,8].

The current literature demonstrates the efficacy of both pharmacotherapy and psychotherapy in treating ADs [9,10]. Cognitive behavioral therapy (CBT) is effective in the treatment of ADs [11]. Nevertheless, face-to-face CBT requires a major input of resources and time, thus limiting the accessibility of such treatment [12,13]. The accessibility and affordability challenge of CBT can be partly resolved by internet-delivered CBT (iCBT) programs [14]. The iCBT treatments are time- and place-independent, are available 24/7, and are less resource-consuming than traditional CBT. Efficacious iCBT techniques are available for different ADs, such as generalized anxiety disorder (GAD), panic disorder (PD), and social anxiety disorder (SAD), as well as for disorders with anxiety as a core symptom, for example obsessive-compulsive disorder (OCD) and posttraumatic stress disorder [15,16].

In routine care, conventional CBT and iCBT demonstrate largely similar effectiveness and acceptability profiles [17,18]. The Cochrane review by Olthuis et al [15] and meta-analysis of Andrews et al [17] demonstrated that disorder-specific iCBT approaches vary in terms of methodology and outcome measures. iCBT clinical trials use disorder-specific rather than transdiagnostic anxiety measurements. However, some of the disorder-specific scales, for example, the Social Phobia Inventory (SPIN) [19] or revised Obsessive-Compulsive Inventory (OCI-R) [20], are relatively long and time-consuming (17 and 42 items, respectively).

Overall Anxiety Severity and Impairment Scale (OASIS) [21] is a brief, easy-to-use, patient-friendly self-report scale to assess severity and impairment associated with anxiety in any anxiety disorder or multiple anxiety disorders [22,23]. OASIS demonstrates good reliability and transcultural validity both in primary care and specialized mental health care settings [24,25]. Given the notorious co-occurrence of 2 or more anxiety disorders [26], OASIS can be especially valuable.

OASIS seems to be a feasible anxiety measure for CBT in the real-world care setting [27]. However, direct extrapolation of the results obtained in face-to-face CBT to iCBT without rigorous scientific evidence would be risky. To our knowledge, OASIS has not been evaluated in iCBT for ADs.

Some studies investigated the relationships between OASIS and other anxiety measurement instruments such as the 7-item Generalized Anxiety Disorder scale and Obsessive-Compulsive Inventory [24,28]. In turn, our study aims to investigate relationships between OASIS and disorder-specific gold-standard instruments in iCBT. We hypothesize that OASIS as an outcome measure has distinct associations with diagnosis-specific scales and could have potential as an alternative to diagnosis-specific measures in some iCBTs and as a transdiagnostic instrument for evaluation of iCBT.

## Methods

### Setting and Study Design

Helsinki University Hospital (HUS) provides original Finnish-language nationwide iCBT (HUS-iCBT) programs for various psychiatric disorders including GAD, SAD, PD, and OCD. The therapy programs are free of charge for patients. Referring patients to the therapy is possible for any licensed physician in Finland. The referring physicians (most often, general practitioners) receive support from web-based instructions but retain overall responsibility throughout the patient’s treatment. For each iCBT program, HUS performs observational, nationwide, open-label, real-world studies, as described by Ritola et al [16]. This study is a cross-diagnostic investigation using specific HUS-iCBT data sets together.

### Participants

The recruitment period in HUS-iCBT for GAD was June 1, 2014, to December 31, 2017, that for OCD was October 1, 2015, to December 31, 2017, that for PD was June 1, 2014, to December 31, 2017, and that for SAD was February 1, 2016, to January 31, 2018.

The inclusion criteria for HUS-iCBT programs were being 18 years of age or older and having a diagnosis of GAD, SP, PD, or OCD verified by the referring physician. Patients with suicidal intentions; current drug misuse; diagnosis of psychotic, bipolar, or serious personality disorder; or impaired cognitive performance due to neurologic or neuropsychiatric disorder were excluded.

Observational studies have been or are currently being conducted for all 4 therapies and included all patients who gave their informed consent, with no additional inclusion or exclusion criteria.

In this study, we performed secondary analyses of the data from those 4 observational studies. No additional informed consent was required.

### Procedure

The HUS-iCBT program for GAD included 12 sessions, that for SAD included 7 sessions, that for PD included 10 sessions, and that for OCD included 10 sessions. Participants were required to complete self-report questionnaires during treatment.

### Therapists

Therapists providing HUS-iCBT were clinical psychologists, psychology students, or nurses with additional therapeutic training employed by HUS. The training of the therapists is described by Ritola et al [16]. The therapists provided support and feedback for the patients throughout the treatment process.

### Measures

#### Overview

Sociodemographic measures (gender and age) were obtained from the therapy referral letters. The longitudinal change in the rating scale scores served as outcomes for each original therapy and original interventional studies. Patients completed all symptom rating scales digitally. All symptom measures are Likert-type scales.

#### HUS-iCBT for GAD

The 7-item Generalized Anxiety Disorder scale (GAD-7) is a self-report scale to measure GAD symptoms based on criteria in the Diagnostic and Statistical Manual for Mental Disorders, Fourth Edition (DSM-IV) [29]. The total score of the GAD-7 ranges from 0 to 21. Pre- and posttreatment values of Cronbach α were .725 and .905, respectively.

The Penn State Worry Questionnaire (PSWQ) is a 16-item self-report scale to assess a core feature of GAD—pathological worry [30]. The total score on the PSWQ ranges from 16 to 80. Pre- and posttreatment values of Cronbach α were .865 and .931, respectively.

#### HUS-iCBT for OCD

The OCI-R is an 18-item self-report scale to measure obsessive-compulsive symptoms on 6 subscales: Obsessing, Washing, Checking, Neutralizing, Ordering, and Hoarding [20]. The total score on the OCI-R ranges from 0 to 72. Pre- and posttreatment values of Cronbach α for the total scale were .796 and .820, respectively.

#### HUS-iCBT for PD

The Panic Disorder Severity Scale (PDSS) is a 7-item self-report scale to assess dimensions of panic disorder, such as frequency of panic attacks, fear and avoidance, and functional and social impairment related to panic disorder [31]. The total score on the PDSS ranges from 0 to 28. Pre- and posttreatment values of Cronbach α were .956 and .963, respectively.

#### HUS-iCBT for SAD

SPIN is a 17-item self-report scale to assess fear, avoidance, and physiological discomfort related to social phobia [19]. The total score on SPIN ranges from 0 to 68. Pre- and posttreatment values of Cronbach α were .875 and .983, respectively.

#### Transdiagnostic Measure

OASIS is a 5-item self-report scale to assess the frequency and severity of anxiety symptoms, anxiety-related avoidance behavior, and decreased functioning at home, work, or school and in social life [21]. The total score on OASIS ranges from 0 to 20. The pretreatment value of Cronbach α for all therapies was .932, and posttreatment values of Cronbach α were .941 for GAD, .945 for OCD, .948 for PD, and 0.954 for SAD. The web-based version of the scale is validated in a clinical sample [32]. In our study, OASIS was used as an additional anxiety measurement instrument in all 4 treatment programs.

### Statistical Analyses

Each treatment program was investigated separately; however, the same statistical methods were used. Total scores were calculated for each symptom scale. Analyses were limited to the comparison of associations and differences between the first (baseline) and last (end point) sessions for each treatment program.

The requirements of parametric tests (homogeneity of error variance and normally distributed error terms) were assessed using graphical methods such as histograms, Q-Q-plots, and scatter plots, as suggested, for example, by Mage [33] and Ernst and Albers [34]. A graphical approach was chosen over normality and homogeneity tests because the sample size was rather large and statistical tests might be overpowered and show discrepancies that are not practically significant. 

Changes in the mean scores of OASIS, GAD-7, PSWQ, OCI-R, PDSS, and SPIN between the first and last sessions were assessed for therapy completers, using a standard univariate ANOVA *F* test.

Bivariate correlation analysis (Pearson product-moment correlation coefficient) was used to estimate correlations of OASIS with GAD-7, PSWQ, OCI-R, PDSS, and SPIN in each session; analysis was performed for each treatment program separately.

A general linear model for repeated measures (mixed models) and interaction analysis were used to investigate the changes and relationships in mean scores of OASIS and GAD-7, PSWQ, OCI-R, PDSS, and SPIN from the first to the last session. Separate models were built for every pair of OASIS and a disorder-specific scale.

The model was chosen on the basis of Bayesian information criteria. The number of bootstrapped random samples was 5000. 

In addition, we calculated the δ variables as a difference between the values of the first and last session of each specific measurement and OASIS. The δ pairs of GAD-7 and OASIS, PSWQ and OASIS, OCI and OASIS, PDSS and OASIS, and SPIN and OASIS were then compared using correlation analyses. The correlations between change scores were analyzed for descriptive purposes only and to show how different variables of interest behave. 

### Ethics Approval

The study protocol and its amendments were approved by the ethics committee of HUS and by pertinent institution authorities (HUS’s chief medical officer; 179/13/03/03/2014). The study was conducted in compliance with the International Council for Harmonization of Technical Requirements for Registration of Pharmaceuticals for Human Use–Good Clinical Practice guidelines, the tenets of the Declaration of Helsinki, and current national regulations. Personal data were pseudonymized. The researchers who had access to the data were unable to reidentify individual patients. In accordance with the Finnish legislation, patients were not compensated for neither their participation in the abovementioned observational studies nor the secondary analyses of the data in this research.

## Results

In the first session, 5504 patients completed OASIS during GAD therapy, 1318 patients during OCD therapy, 1778 patients during PD therapy, and 1636 patients during SAD therapy, and the corresponding figures for the last session were 2714 (49.3%), 604 (45.8%), 401 (22.5%), and 450 (27.5%), respectively.

The mean age of the patients with GAD was 33.6 (SD 11.6) years, 29.9 (SD 9.6) years for those with OCD, 32.7 (SD 11.2) years for those with PD, and 30.5 (SD 10.2) years for those with SAD. The proportion of women in GAD therapy, OCD therapy, PD therapy, and SAD therapy was 76.8% (n=5528), 65.3% (n=1330), 67.6% (n=1856), and 56.6% (n=1645), respectively. The seemingly different preponderance of women by disorder in our population is in line with previously reported figures [35].

In all treatment programs, symptoms of anxiety, measured by both a disorder-specific scale and OASIS, significantly decreased from the first session to the last one (Table 1).

All disorder-specific scales in each treatment program positively correlated with OASIS moderately, strongly, or very strongly in each session when questionnaires were administered (Table 2). The main effect of linear mixed models indicated a clear association between OASIS and disorder-specific scale scores (Table 3). Interaction analysis demonstrated a stable association between the OCI and the PDSS from the first session to the last one, while GAD-7, the PSWQ, and SPIN interrelated with OASIS more strongly at the last session than at the first one (Table 4). Correlation analyses demonstrated significant associations among δ values: OASIS and GAD-7 (δ=0.566, 95% CI 0.523-0.599; *P*<.001), OASIS and PSWQ (δ=0.527, 95% CI 0.495-0.559; *P*<.001), OASIS and OCI (δ=0.392, 95% CI 0.318-0.465; *P*<.001), OASIS and PDSS (δ=0.678, 95% CI 0.604-0.752; *P*<.001), and OASIS and SPIN (δ=0.519, 95% CI 0.447-0.609; *P*<.001).

**Table 1:** Clinical characteristics of patients in internet-delivered cognitive behavioral therapy programs.

Scale		Score, mean (SD)		
		First session		Last session
**Overall Anxiety Severity and Impairment Scale**				
	Generalized anxiety disorder		11.3 (3.4)^a^	8.1 (4.2)
	Obsessive-compulsive disorder		10.9 (3.7)^a^	7.1 (3.8)
	Panic disorder		9.7 (3.9)^a^	6.2 (3.9)
	Social anxiety disorder		11.8 (3.9)^a^	8.5 (4.3)
**Disorder-specific scale**				
	7-item Generalized Anxiety Disorder scale		11.3 (4.5)^a^	6.3 (4.6)
	Penn State Worry Questionnaire		56.1 (11.1)^a^	48.7 (12.5)
	Revised Obsessive-Compulsive Inventory		27.7 (11.9)^a^	14.9 (9.6)
	Panic Disorder Severity Scale		12.3 (5.6)^a^	7.5 (5.4)
	Social Phobia Inventory		40.3 (12.2)^a^	29.0 (13.9)

^a^Paired samples *t* test for evaluating differences between the first and last sessions (*P*<.001).

**Table 2:** Correlations between the Overall Anxiety Severity and Impairment Scale and symptom-specific scales during internet-delivered cognitive behavioral therapy sessions^a^.

Scales			Sessions, δ																		
			1		3		4		5		6		7		8		9		10		12
**Generalized anxiety disorder**																					
	7-item Generalized Anxiety Disorder scale	0.562		N/A^b^		N/A		N/A		0.702		N/A		N/A		N/A		N/A		0.730	
	Penn State Worry Questionnaire	0.738		N/A		N/A		N/A		0.524		N/A		N/A		N/A		N/A		0.624	
**Obsessive-compulsive disorder**																					
	Revised Obsessive-Compulsive Inventory	0.448		N/A		0.560		N/A		N/A		N/A		N/A		N/A		0.595		N/A	
**Panic disorder**																					
	Panic Disorder Severity Scale	0.693		0.802		N/A		0.803		N/A		N/A		0.860		0.845		0.631		N/A	
**Social anxiety disorder**																					
	Social Phobia Inventory	0.578		0.695		N/A		0.700		N/A		0.703		N/A		N/A		N/A		N/A	

^a^For all correlations, *P*<.001 (Pearson correlation analysis).

^b^N/A: not applicable.

**Table 3:** Associations between the Overall Anxiety Severity and Impairment Scale and disorder-specific scales (linear mixed model analyses)—main effect.

Variable	*F* test (*df*; SE)	95% CI	*t* test (*df*)	*P* value
7-item Generalized Anxiety Disorder scale	0.8 (5360.9; 0.01)	0.7-0.8	45.2 (5759.3)	<.001
Penn State Worry Questionnaire	1.7 (5116.5; 0.04)	1.7-1.8	45.2 (7906.4)	<.001
Revised Obsessive-Obsessive Inventory	1.3 (1124.5; 0.1)	1.1-1.4	14.8 (1813.5)	<.001
Panic Disorder Severity Scale	1.0 (780.4; 0.4)	0.9-1.1	24.4 (1613.5)	<.001
Social Phobia Inventory	2.2 (1081.6; 0.1)	1.9-2.4	22.8 (1907.7)	<.001

**Table 4:** Interaction analysis of the associations between the Overall Anxiety Severity and Impairment Scale and disorder-specific scales (linear mixed model analyses)—estimates of fixed effects^a^.

Variable	*F* test (*df*; SE)	95% CI	*t* test (*df*)	*P* value
7-item Generalized Anxiety Disorder scale	–0.06 (4345.8; 0.02)	–0.109 to –0.017	–2.7 (4228.2)	.007
Penn State Worry Questionnaire	–0.52 (4270.8; 0.04)	–0.620 to –0.437	–11.3 (4585.1)	<.001
Revised Obsessive-Obsessive Inventory	0.09 (822.9; 0.09)	0.090 to 0.277	1.0 (899.6)	.32
Panic Disorder Severity Scale	–0.02 (596.6; 0.04)	–0.108 to –0.065	–0.5 (660.8.5)	.63
Social Phobia Inventory	–0.39 (862.1; 0.10)	–0.596 to –0.187	–3.8 (896.5)	<.001

^a^Estimates are interactions between the scale score and assessment at the first session, thus indicating the difference in the associations of scales between the first and the last sessions.

## Discussion

### Principal Findings

To our knowledge, this is the first study investigating the feasibility of OASIS as an outcome measure in iCBT for ADs. In all iCBT programs at the HUS included in this study (those for GAD, PD, SAD, and OCD), OASIS mean scores significantly decreased from baseline to the end point, as did those of all disorder-specific symptom severity measures. OASIS had clear and relatively stable associations with disorder-specific symptom measures. According to estimates of fixed effects in mixed models, associations of OASIS with OCI-R and PDSS were significant and stable within the treatment programs. By contrast, relationships of OASIS with SPIN, PSWQ, and GAD-7 did not demonstrate such stability. Associations between OASIS and SPIN, PSWQ, and GAD were stronger at the end of treatment than at the beginning. Thus, in SAD and GAD therapies, OASIS appears to be differentially sensitive to the severity of disorder-specific symptoms across the treatment phase. However, the lower first-session associations between OASIS and SPIN, PSWQ, and GAD-7 were 69%-93% of the higher last-session associations, suggesting moderate stability of the association from first to the last session. Excluding PSWQ, which targets worry rather than anxiety, the lower first-session association was 82% (SPIN) or 93% (GAD-7) of the higher last-session association. Thus, coherent interrelation of the OASIS and disorder-specific scales at the end of treatment indicates the capability of OASIS, especially as a follow-up instrument.

For face-to-face treatments, OASIS is a valid transdiagnostic outcome measurement instrument in both nonclinical and clinical samples [36,37] in different therapeutic interventions for ADs. Our results suggest that OASIS might have potential as a transdiagnostic outcome measure in iCBT as well.

Recent studies demonstrate that in iCBT, patient-reported lack of sufficient time or being too busy was associated with decreased user satisfaction and weakened adherence to the iCBT [38,39]. In addition, extensive text content of the therapy modules (including long questionnaires), requiring high levels of concentration and reading skills, appears to be an obstacle to successful treatment [40]. Thus, OASIS as a short, easy-to-use instrument may offer a worthwhile option to enhance adherence and, thereby, the overall effectiveness of internet-based therapy for ADs. Overall, we assume that OASIS can become a worthwhile alternative to the disorder-specific scales used in this study. The specificity of disorder-related scales should be weighed against the lightness and easiness of OASIS when choosing measures for iCBT for ADs on a case-by-case basis, depending on concrete needs of researchers or practitioners.

### Strengths and Limitations

This study was conducted on a nationwide scale with a large number of participants, and the data were gathered in a previously underexplored routine clinical practice setting.

The absence of a control with clinician-applied measures might be seen as a limitation. However, all scales with which OASIS was compared are valid, reliable, and widely used psychometric measures. Moreover, it should be recognized that the use of self-rating scales is the only realistic form of outcome measurement in large-scale iCBT programs in busy routine care with high patient volumes [41].

Our study did not apply gold-standard measures of functional impairment, and the feasibility of the impairment subscale of OASIS could not, thus, be separately elucidated.

Our study included HUS-iCBT programs for many but not all ADs. For instance, specific phobias, agoraphobia, and separation anxiety disorders were not included. This leaves the question of feasibility of OASIS for iCBT for these disorders unresolved.

### Future Prospects

OASIS includes items for assessment of both symptom severity and anxiety-related functional impairment. Gold-standard functional impairment measures should be used in future studies to assess whether OASIS in iCBT could replace not only other symptom severity measures but also separate functional impairment scales. If this should prove to be the case, use of OASIS could facilitate the measurement battery in a range of diagnosis-specific and plausibly even transdiagnostic iCBT programs for ADs.

Future research is needed to evaluate the feasibility of OASIS for iCBT programs for ADs not included in our study. Furthermore, more research is required to assess the utility of OASIS for between-disorder comparison of the effectiveness of iCBT for various ADs. Psychometric properties of OASIS across different iCBTs could be subsequently investigated.

### Conclusions

OASIS demonstrates clear and relatively stable associations with disorder-specific symptom measures in iCBT for a range of ADs. Considering the benefits of OASIS as a short, easy-to-use self-rating scale, it might have an implementation as an outcome measurement instrument for disorder-specific and possibly also transdiagnostic iCBT programs for ADs in regular clinical practice. Further research is needed to elucidate the feasibility of OASIS for iCBT for ADs other than GAD, PD, SAD, and OCD and to gain an overview of the use of OASIS’ functional impairment subscale.

## Abbreviations

AD: anxiety disorder
CBT: cognitive behavioral therapy
DSM-IV: Diagnostic and Statistical Manual for Mental Disorders, Fourth Edition
GAD: generalized anxiety disorder
GAD-7: 7-item Generalized Anxiety Disorder scale
HRQL: health-related quality of life
HUS: Helsinki University Hospital
iCBT: internet-delivered cognitive behavioral therapy
OASIS: Overall Anxiety Severity and Impairment Scale
OCD: obsessive-compulsive disorder
OCI-R: revised Obsessive-Compulsive Inventory
PD: panic disorder
PDSS: Panic Disorder Severity Scale
PSWQ: Penn State Worry Questionnaire
SAD: social anxiety disorder
SPIN: Social Phobia Inventory

## Data Availability

The data used in this study are not publicly available due to the national and international privacy regulations concerning sensitive health care data.
